# 2,1,3-Benzothiadiazole Small Donor Molecules: A DFT Study, Synthesis, and Optoelectronic Properties

**DOI:** 10.3390/molecules26051216

**Published:** 2021-02-24

**Authors:** Dorota Zając, Damian Honisz, Mieczysław Łapkowski, Jadwiga Sołoducho

**Affiliations:** 1 Faculty of Chemistry, Wrocław University of Science and Technology, Wybrzeże Wyspiańskiego 27, 50-370 Wrocław, Poland; jadwiga.soloducho@pwr.edu.pl; 2 Faculty of Chemistry, Silesian University of Technology, Strzody 9, 44-100 Gliwice, Poland; Damian.Honisz@polsl.pl (D.H.); Mieczyslaw.Lapkowski@polsl.pl (M.Ł.); 3 Centre of Polymer and Carbon Materials, Polish Academy of Science, 34 Curie Sklodowska Str., 41-819 Zabrze, Poland

**Keywords:** benzothiadiazole, small molecules, organic optoelectronic, Stille, Suzuki reaction, conjugated polymers, luminescence materials

## Abstract

We herein report the design and synthesis of small-donor molecules, 2,1,3-benzothiadiazole derivatives (**2a**–**d**), by Stille or Suzuki reaction. The synthesized compounds were characterized by spectroscopic and electrochemical methods. The compounds **2a**–**d** absorb the light in a wide range (the UV-green/yellow light (**2c**)) and emit from green to red/near IR light (**2c**). Furthermore, these compounds show a narrow energy gap (1.75–2.38 eV), and high Ea values increasing for polymers, which prove their electron-donating nature and semiconductor properties. The measurements were enhanced by theoretical modeling.

## 1. Introduction

Organic semiconductors are currently used in many fields of science, e.g., organic light-emitting diode (OLED), solar cells, transistors, molecular imaging, and sensors [1,2,3,4,5,6,7]. Due to their conductive and optical properties, the possibility of easy modification, flexibility, and low production costs, they displace their inorganic counterparts. A recent breakthrough in the performance of organic semiconductor devices has been achieved by developing non-fullerene acceptors (NFAs) that can overcome the drawbacks of fullerenes and have the advantages of high absorption rates, readily tunable optical and electronic properties, and increased solubility [8,9]. Using several variants of the semiconductor structural systems, the properties of the entire system are adjusted, e.g., donor–acceptor–donor (D–A–D) molecules [10,11]. An example is the use of small molecules, which are currently being intensively studied due to the shortage of n-type conjugated polymer (n-CP) materials as an acceptor in the active layer and a p-type conjugated polymer (p-CP) as a donor, especially in organic solar cells. Currently, n-CP focuses primarily on perylene diimide, naphthalene diimide, pyridine derivatives, and p-CP containing thiophene and dithienosilole [12,13,14,15,16,17]. However, many of them suffer from some significant weaknesses, such as a poor absorption coefficient and excessively strong crystallinity and stacking, which lead to a limited photocurrent and poor separation in the active layers [18,19,20,21]. To overcome the unfavorable properties of some conjugated polymers (CPs), small molecules are being researched, considering their advantages of easy purification, a narrow bandgap, strong absorption, higher electron mobility, well-defined chemical structures, and good photovoltaic performance reproducibility without batch-to-batch variation [22,23,24,25,26,27,28,29].

**Scheme 1:**
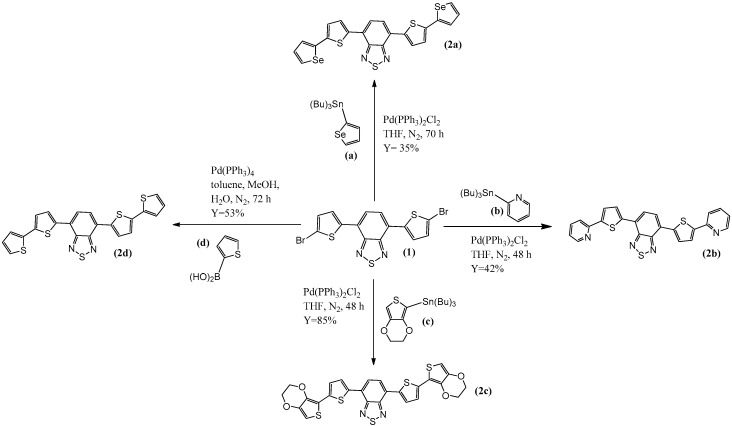
Synthesis routes of the benzothiadiazole derivatives (**2a**–**d**).

Herein, to further explore and adjust the properties of novel small molecular donors, 4,7-bis(5-(selenophen-2-yl)thiophen-2-yl)benzothiadiazole (**2a**) and 4,7-bis(5-(pyridin-2-yl)thiophen-2-yl)benzothiadiazole (**2b**) were designed and synthesized according to our previously reported synthesis of 4,7-bis(5-(3,4-ethylenedioxythiophene)thiophen-2-yl)benzothiadiazole (**2c**), as depicted in Scheme 1 [30]. The benzothiadiazole derivatives contained the D–A–D structural motif, based on selenophene, pyridine or 3,4-ethylenedioxythiophene (donor), thiophene (bridge), and benzothiadiazole (BTD, acceptor) from commercially available precursors via only one step. To better understand the dependence of optoelectronic properties on the structure, 4,7-di([2,2′-bithiophen]-5-yl)benzothiadiazole (**2d**) was also synthesized to compare the effect of thiophene as a substituent in comparison to other groups [31].

## 2. Results

### 2.1. Synthesis

The synthetic routes and chemical structures of the **2a**–**d** molecules are depicted in Scheme 1. The target small molecules **2a**–**c** were obtained through a Stille coupling reaction, and compound **2d** was obtained through a Suzuki coupling reaction. Selenophene and 3,4-ethylenedioxythiophene were monostannylated to 2-(tributylstannyl)selenophene or 2-(tributylstannyl)-3,4-ethylenedioxythiophene in 99% yield with SnMe_3_Cl in THF at −80 °C. All the synthesized compounds were further purified by using a column chromatography technique. The molecular structures of these target compounds and their intermediates were established by using different spectroscopic tools.

### 2.2. Theoretical Studies

**Figure 1:**
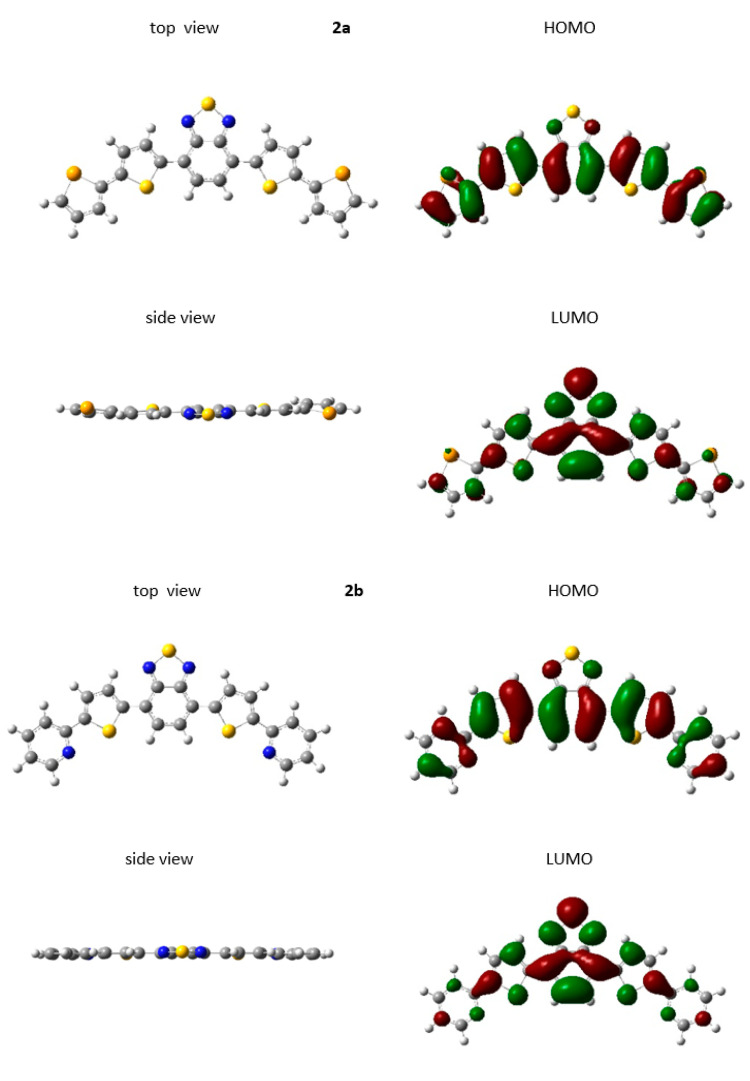

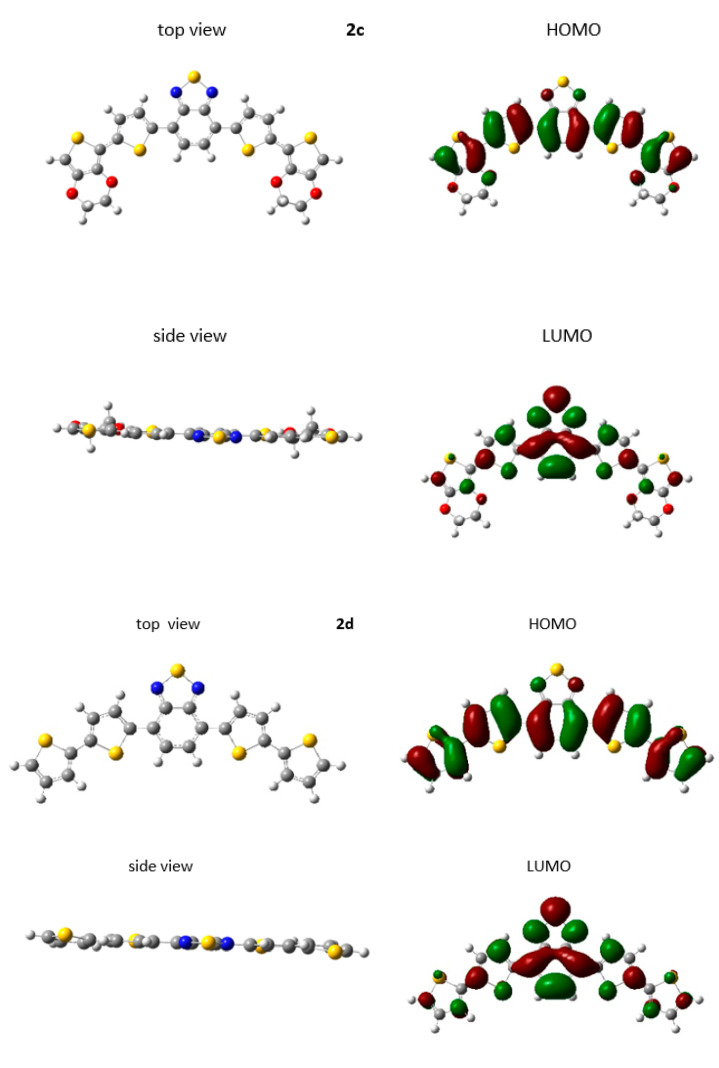
Optimized structures and Frontier molecular orbitals of **2a**–**d**.

**Figure 2:**
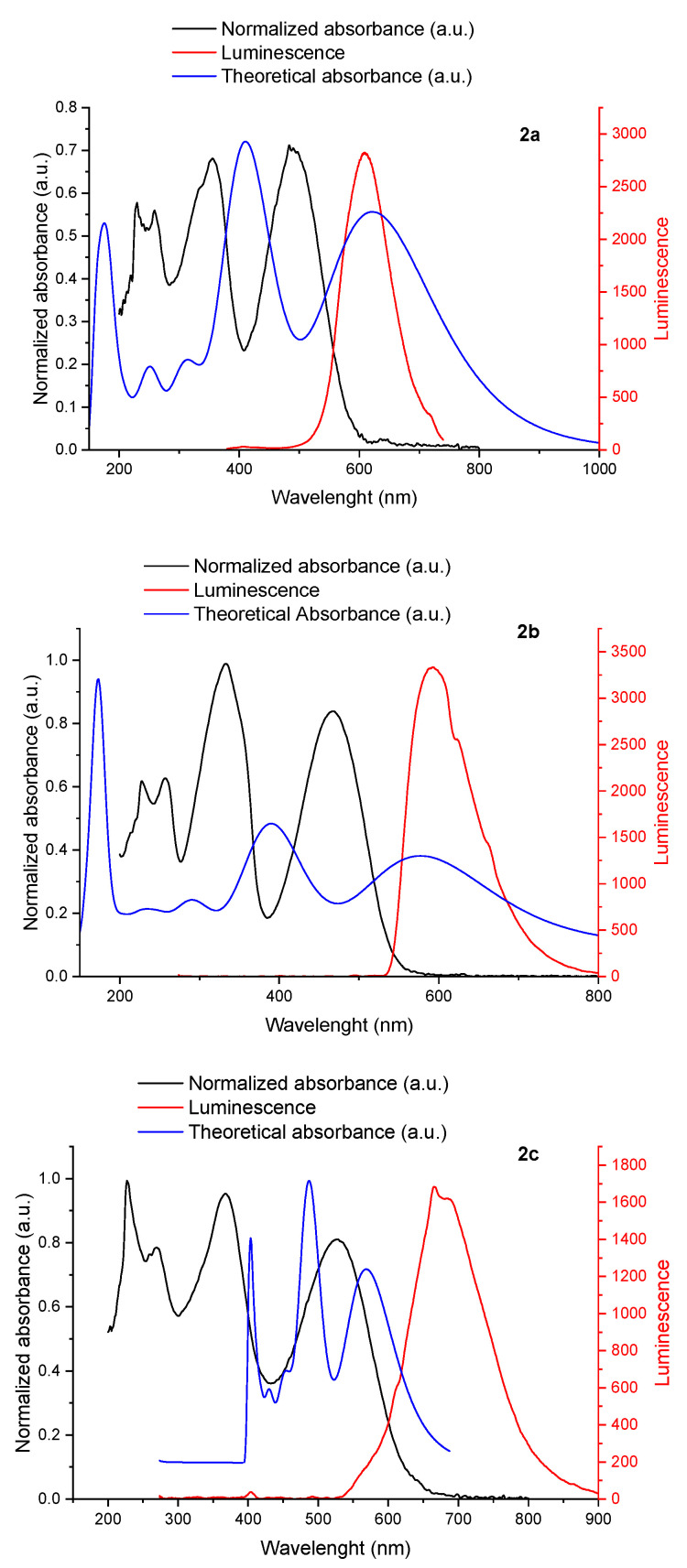

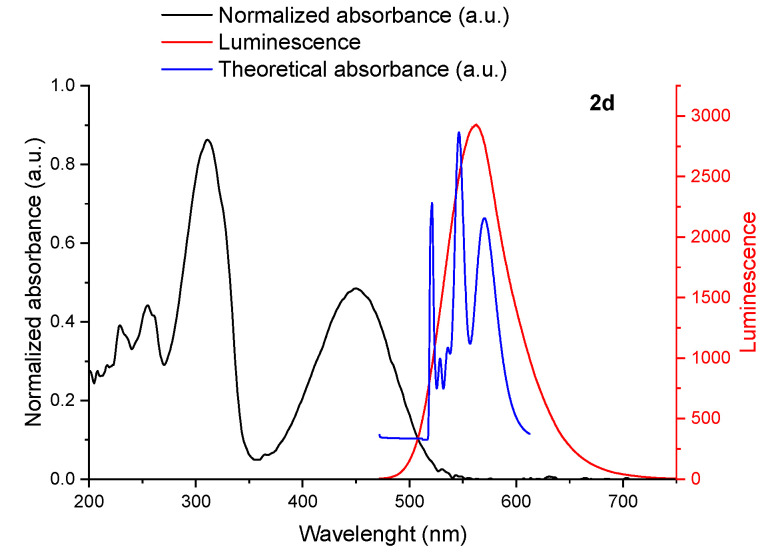
UV-vis experimental absorption (black), theoretical absorption (blue, in vacuum), and emission (red) spectra of compounds **2a**–**d** in dichloromethane (DCM).

Figure 1 shows the optimized structures and frontier molecular orbitals of **2a**–**d**. The HOMO energy level was found to spread throughout the molecules, whereas LUMO was localized on the benzothiadiazole moiety. The HOMO and LUMO energy levels of **2a**–**d** are stated in Table 1. The observation, however, should be treated with caution, since the orbital energies are basis set dependent. However, these values are reasonably close to both calculated and experimental ionization energies (Table 1). The efficient small molecule donors should have the HOMO energy levels around −5.2 eV to ensure acceptable device open circuit voltages [32]. All compounds, except **2c**, fulfill this condition. The lowest HOMO orbital has **2b** (−5.24 eV), then **2d** (−5.12 eV), **2a** (−5.12 eV), and **2c** (−4.78 eV). Moreover, the value of the ionization potential suggests the donor properties of the compounds (ionization potential (IP) < 5.7 eV) (Table 1) [33]. Compound **2c** has the lowest IP_ad_^cal^ (5.7 eV) compared to compounds **2a** (6.07 eV), **2d** (6.08 eV), and **2b** (6.25 eV), and therefore has the strongest donor properties. These results differ by approximately 0.5 eV (**2d** by 0.2 eV) from the experimental results, respectively: 5.24 eV (**2c**), 5.60 eV (**2a**), 5.86 eV (**2d**), and 5.76 eV (**2b**). Furthermore, the structure of compounds **2a**–**d** is planar, which ensures close intermolecular contacts and high charge carrier mobilities [32]. Figure 2 shows the experimental and calculated UV-vis absorption and photoluminescence spectra of **2a**–**d**. The corresponding data are summarized in Table 2. It is observed that the λ_max_^cal^ of all the structures corresponds to HOMO → LUMO transitions (range in yellow and red light) and to HOMO→LUMO + 1 transitions (in the violet range). The calculated λ_max_ value of molecule **2a** shows the broadest wavelength with maximum absorption at 413 and 623 nm, which is red-shifted by 58 and 140 nm when compared with experimental results. The theoretical UV-vis spectra of **2b**, **c**, and **d** are also red-shifted (110–170 nm) in comparison with experimental results. Moreover, **2c** and **2d** show similar absorption behavior, but **2c** shows broader and red-shifted spectra with the maximum of absorbance at 418 and 652 nm. The compounds **2a**–**d** have high oscillator strength (f), which corresponds to the appearance of a high experimental absorption coefficient (Table 2).

### 2.3. Photophysical Studies

The optical properties of synthesized compounds **2a**–**d** were studied by UV-vis and fluorescence spectroscopy. The UV-vis absorption and emission spectra of compounds **2a**–**d** in dichloromethane (DCM) are shown in Figure 2, and the corresponding data are summarized in Table 2. Compounds **2a**–**d** show two absorption peaks at 311–370 nm due to the overlap of the n-π* and π-π* transition, whereas absorption maxima at 452–526 nm are due to a push–pull system, which allows for intramolecular electron density transfer from donor unit to acceptor. Compounds **2a**, **b**, and **d** show luminescence in the range of light from green to red light, with the maximum of emission at 609, 592, and 563 nm, respectively, while compound **2c** is batochromically shifted to near IR, with the maximum of emission at 667 nm. This red shift in the emission spectrum of **2c**, as compared to **2a**, **b**, and **d**, can be explained in terms of the stronger donor character of ethylenedioxythiophene.

### 2.4. Electrochemical Properties

**Figure 3:**
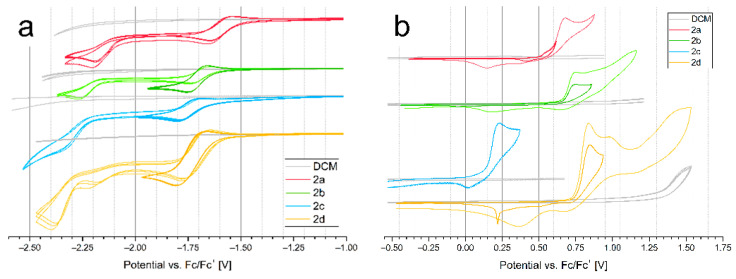
(**a**) The Cyclic Voltammetry (CV) of reduction of monomers versus ferrocene redox couple, concentration 1 mmol/dm^3^ in DCM, 100 mV/s and background (gray line); (**b**) the CV of oxidation of monomers versus ferrocene redox couple, concentration 1 mmol/dm^3^ in DCM, 100 mV/s and background (gray line).

During the electrochemical oxidation, an irreversible process of polymerization occurs for all monomers. First, Cyclic Voltammetry (CV) scans in the anodic range are presented in Figure 3b. The oxidation potential of **2c** was much lower than that of compounds **2a**, **2b**, and **2d**, which subsequently oxidized within a narrow range. This trend for changing substituents is consistent with the literature [34,35] and with calculated values of HOMO. During reverse polarization, reduction of oligomers occurs. For **2d**, the first oxidation scan’s sharp reduction peak suggests dimer formation.

CV of three reduction scans is presented in Figure 3a. The similar reduction potential of all compounds is caused by the connection of the changing donor unit by the thiophene β position and its long distance from the benzothiadiazole unit, where the LUMO is localized. Differently calculated values of LUMO vary between compounds. That indicates that the communication between the D-A parts is slightly overestimated by the density functional theory (DFT), probably by the ideal planarity of the optimized structure. Additionally, the absolute values of LUMO are significantly lower than Electron Affinities, which is usual for the LUMO calculated by the DFT method [36]. Reduction potentials of first quasi-reversible peaks are slightly less electronegative than pure dithienylbenzothiadiazole (−1.74 V at onset) [34]. Monomer electrochemical results are summarized in Table 1. All compounds show a narrow band gap (1.75–2.38 eV). The narrowest band gap (ΔE*_g_*^el^) has **2c** (−1.75 eV), then **2a** (2.01 eV), **2b** (2.28 eV), and **2d** (2.38 eV). These results correlate well with the theoretical values (E_g_^cal^). The largest difference between the experimental and theoretical results was observed for compound **2c** (0.44 eV), and the smallest for compound **2d** (0.09 eV).

**Figure 4:**
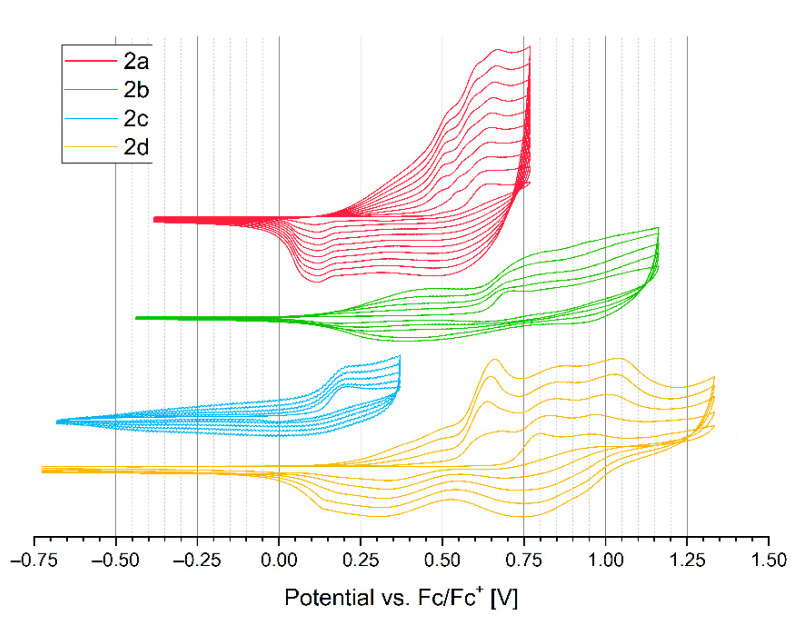
Cyclic polymerization versus ferrocene redox couple, concentration 1 mmol/dm^3^ in DCM, 100 mV/s.

**Figure 5:**
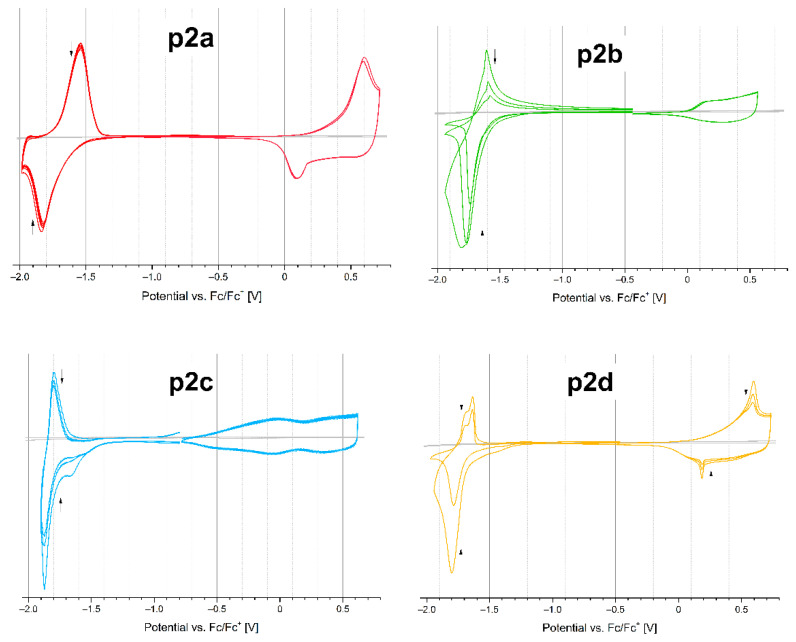
The CV of polymer films **p2a**–**d** on Pt wire versus ferrocene redox couple in DCM, 100 mV/s and background (gray line).

Electropolymerization of investigated compounds (Figure 4) forms insoluble films on platinum wire. Polymer CV was conducted under the same conditions as monomers. Three scans of separately registered oxidation and reduction are presented in Figure 5. Polymers **p2b**, **p2c**, and **p2d** undergo rapid degradation while reducing **2a** also degrades, but slower. During oxidation, **p2b** and **p2c** remain stable, and **p2a** is stable after the first scan. Only **p2d** clearly degraded under oxidation. A high drop in **p2c** oxidation onset in comparison with **2c** (0.83 V) is characteristic for 3,4-ethylenedioxythiophene (EDOT)-ended monomers and results in a very low bandgap (0.72 V) [37]. The oxidation potential of polymers **p2d** and **p2b** is similar, but the potential of the latter is lower, like for the corresponding monomers. However, **p2a**’s oxidation potential decreased after polymerization by only approximately 0.33 V. All results for polymers are shown in Table 3.

Moreover, compound **2c** was electropolymerized, and the obtained film, poly(4,7-bis(5-(3,4-ethylenedioxythiophene)thiophen-2-yl)benzothiadiazole), served as a matrix for an enzyme, horseradish peroxidase (HRP), for 17β-estradiol detection in an electrochemical biosensor [30]. The detection limit for 17β-estradiol was set to 105 nM, the sensitivity of the proposed biosensor was found to be 1.16 × 10^−4^ A·μM^−1^·cm^−2^, and the lifetime of the system can be determined for 5 weeks. Compound **2b** also was used in an amperometric, tyrosinase-based biosensor for epinephrine detection [38]. The sensitivity of the proposed biosensor was found to be 3.08 × 10^−7^ A·µM^−1^·cm^−2^. Poly(**2c**) and poly(**2b**) serve as electron mediators to improve the flow of electrons between the enzyme’s active center and the electrode surface, and they act as a transducer during the transfer of electric charge [30,38]. Both of these sensors show a good sensitivity, which confirms the semiconductor nature of the obtained compounds.

## 3. Materials and Methods

### 3.1. Computational Details

The theoretical studies were performed by applying the density functional theory (DFT) method [39]. The calculations were performed utilizing the B3LYP functional [40,41,42] and the standard cc-pVDZ atomic basis set [43]. The basis set was adopted based on the former experience [44,45,46]. This basis provides orbital HOMO energies reasonably reproducing directly calculated ionization energies (Table 1). Optical transition of all compounds was studied by conducting Time-dependent density-functional theory (TD-DFT) computations [47]. To simulate the UV-vis absorption, spectra and oscillator strength were estimated at their ground-state optimized geometries for a maximum of 200 excited states. All the calculations were carried out using the Gaussian16 suite of codes [48]. The computational resources were provided by the Wroclaw Centre for Networking and Supercomputing (http://wcss.pl). The molecular graphics were produced by applying the GausView program (Gaussian, Inc. Wallingford, Connecticut, USA) [49].

### 3.2. Chemistry

*n*-Butyllithium (2.5 M in hexane), trimethyltin chloride (1.0 M in THF), 2-(tributylstannyl)pyridine (85%), 3,4-ethylenedioxytiophene (97%), selenophene (97%), 2-thienylboronic acid (95%), bis(triphenylphosphine)palladium (II) dichloride (98%), tetrakis(triphenylphosphine)palladium (0) (99%), and 4,7-bis(5-bromothiophen-2-yl)benzothiadiazole (99%) were purchased from Sigma Aldrich. Anhydrous potassium carbonate (99%) was received from Chempur. Anhydrous tetrahydrofuran, toluene, and methanol were purchased from POCH. Tetrahydrofuran was dried over Na/benzophenone ketal before use. Other commercially available substances and reagents were used without any prior purification. Preparative column chromatography was performed on the glass column with Acros Organics silica gel for chromatography, 0.035–0.075 mm, 60 Å. ^1^H-NMR and ^13^C-NMR spectra were recorded in deuterated chloroform (CDCl_3_) on Brüker Avance III 400 MHz Instruments or on Bruker Avance II 600 Instruments, respectively. Chemical shifts were locked to chloroform δH 7.26 (s) and δC 77.16 (t) signals. The molecular weights of the products were determined using a Brüker micrOTOF-Q spectrometer, FWHM-17500, 20 Hz (Billerica, MA, USA). The percentage composition of the elements was measured on a vario EL cube Analyzer from Elementar Americas (Ronkonkoma, New York, NY, USA).

#### 3.2.1. Preparation of 4,7-bis(5-(selenophen-2-yl)thiophen-2-yl)benzothiadiazole (**2a**)

To a mixture of 4,7-bis(5-bromothiophen-2-yl)benzothiadiazole (**1**) (1.00 g, 2.18 mmol) and 2-(tributylstannyl)selenophene (2.02 g, 4.80 mmol) in anhydrous THF (80 mL), bis(triphenylphosphine)palladium(II) dichloride (Pd(PPh_3_)_2_Cl_2_) (0.31 g, 0.436 mmol) was added at room temperature under nitrogen atmosphere. The resulting mixture was refluxed with stirring for 70 h. Then, the reaction mixture was concentrated under reduced pressure, diluted with water, and extracted with EtOAc. The extract was washed with brine, dried over MgSO_4_, and concentrated. The residue was purified by silica gel column chromatography (hexane-EtOAc) to give **2a** (0.430 g, 35%) as a brown solid.

Mp: 114 °C

^1^H-NMR (400 MHz, CDCl_3_), δ (ppm): δ 8.11 (dd, *J_1_* = 4.0 Hz, *J_2_* = 5.2 Hz, 1H), 8.02 (d, *J* = 4.0 Hz, 1H), 7.92 (dd, *J_1_* = 5.2 Hz, *J_2_* = 5.6 Hz, 1H), 7.87–7.83 (m, 3H), 7.46–7.44 (m, 3H), 7.29–7.27 (m, 1H), 7.21–7.19 (m, 2H).

^13^C-NMR (151 MHz, CDCl_3_), δ (ppm): δ 152.22, 140.54, 139.21, 138.34, 132.37, 130.83, 128.92, 128.09, 127.72, 127.35.

MS (*m/z*): [M]^+^ 559.1972

Elemental analysis: calc. (%) C:47.32; H:2.17; N:5.02; S:17.23; found: C:47.27; H:2.22; N:5.00; S:17.18.

#### 3.2.2. Preparation of 4,7-bis(5-(pyridin-2-yl)thiophen-2-yl)benzothiadiazole (**2b**)

To a mixture of 4,7-bis(5-bromothiophen-2-yl)benzothiadiazole (**1**) (1.00 g, 2.18 mmol) and 2-(tributylstannyl)pyridine (1.77 g, 4.80 mmol) in anhydrous THF (80 mL), bis(triphenylphosphine)palladium(II) dichloride (Pd(PPh_3_)_2_Cl_2_) (0.31 g, 0.436 mmol) was added at room temperature under nitrogen atmosphere. The resulting mixture was refluxed with stirring for 70 h. Then, the reaction mixture was concentrated under reduced pressure, diluted with water, and extracted with EtOAc. The extract was washed with brine, dried over MgSO_4_, and concentrated. The residue was purified by silica gel column chromatography (hexane-EtOAc) to give **2b** (0.417 g, 42%) as a red solid.

Mp: 151–152 °C

^1^H-NMR (400 MHz, CDCl_3_), δ (ppm): δ 8.64 (d, *J* = 4.8 Hz, 2H), 8.18 (t, *J* = 3.4 Hz, 2H), 7.95 (t, *J* = 9.4 Hz, 2H), 7.82–7.78 (m, 6H), 7.15 (t, *J* = 3.4 Hz, 2H).

^13^C-NMR (151 MHz, CDCl_3_), δ (ppm): δ 152.57, 152.32, 152.18, 152.04, 149.32, 141.36, 140.53, 137.05, 132.45, 130.89, 130.72, 128.91, 128.80, 127.33, 126.04, 125.78, 125.68, 125.34, 125.19, 125.08.

MS (*m/z*): [M]^+^ 455.0434

Elemental analysis: calc. (%) C:63.41; H:3.10; N:12.32; S:21.16; found: C:63.36; H:3.03; N:12.26; S:21.11.

#### 3.2.3. Preparation of 4,7-bis(5-(3,4-ethylenedioxythiophene)thiophen-2-yl)benzothiadiazole (**2c**)

To a mixture of 4,7-bis(5-bromothiophen-2-yl)benzothiadiazole (**1**) (1.00 g, 2.18 mmol) and 2-(tributylstannyl)-3,4-ethylenedioxythiophene (2.07 g, 4.80 mmol) in anhydrous THF (80 mL), bis(triphenylphosphine)palladium(II) dichloride (Pd(PPh_3_)_2_Cl_2_) (0.306 g, 0.436 mmol) was added at room temperature under nitrogen atmosphere. The resulting mixture was refluxed with stirring for 48 h. Then, the reaction mixture was concentrated under reduced pressure, diluted with water, and extracted with EtOAc. The extract was washed with brine, dried over MgSO_4_, and concentrated. The residue was purified by silica gel column chromatography (hexane-EtOAc) to give **2c** (1.075 g, 85%) as a purple solid.

According to our previous work [30].

Mp: 143–145 °C

^1^H-NMR (400 MHz, CDCl_3_), δ (ppm): δ 8.07 (d, *J* = 4.0 Hz, 2H), 7.83 (s, 2H), 7.30 (d, *J* = 4.0 Hz, 2H), 6.27 (s, 2H), 4.41–4.39 (m, 4H), 4.28–4.26 (m, 4H).

^13^C-NMR (151 MHz, CDCl_3_), δ (ppm): δ 150.15, 146.93, 132.46, 132.24, 130.89, 130.52, 128.80, 128.48, 123.76, 114.70, 101.66, 90.06, 68.72.

MS (*m/z*): [M]^+^ 580.9799

Elemental analysis: calc. (%) C:53.77; H:2.78; N:4.82; S:27.61; found: C:53.75; H:2.75; N:4.79; S:27.58.

#### 3.2.4. Preparation of 4,7-di([2,2′-bithiophen]-5-yl)benzothiadiazole (**2d**)

To a mixture of 4,7-bis(5-bromothiophen-2-yl)benzothiadiazole (**1**) (1.00 g, 2.18 mmol), 2-thienylboronic acid (0.64 g, 5.02 mmol) and potassium carbonate (0.90 g, 6.54 mmol) in toluene (30 mL), MeOH (6 mL), and water (6 mL), tetrakis(triphenylphosphine)palladium(0) (Pd(PPh_3_)_4_) (0.126 g, 0.109 mmol) was added at room temperature under nitrogen atmosphere. The resulting mixture was refluxed with stirring for 72 h. Then, the reaction mixture was concentrated under reduced pressure, diluted with water, and extracted with EtOAc. The extract was washed with brine, dried over MgSO_4_, and concentrated. The residue was purified by silica gel column chromatography (hexane-EtOAc) to give **2d** (0.54 g, 53%) as a red solid. According to [31].

Mp: 189–191 °C

^1^H-NMR (400 MHz, CDCl_3_), δ (ppm): δ 8.12 (dd, *J_1_* = 0.8 Hz, *J_2_* = 3.6 Hz 2H), 7.80 (t, *J* = 4.0 Hz, 4H), 7.70–7.68 (m, 2H), 7.53–7.51 (m, 2H), 7.15 (d, *J* = 4.0 Hz, 2H).

^13^C-NMR (151 MHz, CDCl_3_), δ (ppm): δ 152.12, 140.54, 132.46, 130.90, 130.76, 128.85, 128.14, 127.72, 127.35, 125.69, 125.17.

MS (*m/z*): [M]^+^ 463.9600

Elemental analysis: calc. (%) C: 56.87; H: 2.60; N: 6.03; S: 34.50; found: C:56.82; H:2.56; N:5.95; S:34.46.

### 3.3. Optical Measurements

UV-vis spectra were recorded on the Spectroquant Pharo 300 spectrophotometer (Merck, Darmstadt, Germany). The luminescence spectra were recorded on the Hitachi F-2500 fluorescence spectrophotometer (Hitachi High Technologies America, Inc., Pleasanton, CA, USA).

### 3.4. Cyclic Voltammetry

For the cyclic voltammetry (CV) experiments, a three-electrode glass cell was used with a platinum wire as a working electrode, a platinum wire spiral as a counter electrode, and a silver wire reference using 0.1 M NBu_4_PF_6_ (TCI Europe) electrolyte solution in dichloromethane (DCM) (Sigma-Aldrich (Poznan, Poland), Chromasolv, HPLC). The potential sweeps were controlled by a MetrohmAutolab PGSTAT 100 N potentiostat. The potential of the silver electrode was determined using a ferrocene redox couple (Fc/Fc^+^) for each measurement set, under the same conditions as the measured samples. The solutions were de-aerated with argon before, and argon kept flowing into the cell, above the solution surface, during measurements. A concentration of 1 mmol/dm^3^ of the monomers was used for both the measurements and polymerization.

## 4. Conclusions

In summary, we have designed and synthesized a series of benzothiadiazole derivatives as donor small molecules with good yield. In the case of the **2c** derivative, we obtained up to 85% yield, which gives an excellent result in the Stille reaction. The **2a** and **2c** compounds absorb the light in the UV-yellow range, and the **2b** and **2d** compounds in the UV-green range. Moreover, these compounds emit in a very wide range (**2a**, **b**, and **d** from green to red and **2c** from green to near IR); therefore, these compounds are very promising optoelectronics materials, e.g., in White OLED (WOLED). In addition, they show a narrow energy gap (1.75–2.38 eV), especially as polymer films (0.72–1.64 eV), and low IP values decreasing for polymers (5.24–5.86 eV for monomers and 4.41–5.27 eV for polymers), which proves their electron-donating nature and semiconductor properties. Furthermore, compound **2c** and **2b** served as a conductive matrix for an enzyme in biosensors.

## Figures and Tables

**Table 1:** Redox properties of monomers. E^ox^_onset_—oxidation peak onset; E^red^_onset_—reduction peak onset; IP—ionization potential, estimated from the equation IP[eV] = |e|5.1 + E^ox^_onset_[V]; EA—electron affinity, estimated from the equation EA[eV] = |e|5.1 + E^red^_onset_[V]; ΔE_g_^el^—electrochemical bandgap from the equation ΔE_g_^el^ = IP − EA; IP_ver_^cal^—calculated vertical ionization potential; IP_ad_^cal^—calculated adiabatic ionization potential; E_g_^cal^—calculated bandgap; cal—calculated.

Compound	E^ox^_onset_ (V)	E^red^_onset_ (V)	IP (eV)	IP_ver_^cal^ (eV)	IP_ad_^cal^ (eV)	EA (eV)	HOMO^cal^ (eV)	LUMO^cal^ (eV)	E_g_^cal^ (eV)	ΔE_g_^el^ (eV)
**2a**	0.50	−1.51	5.60	6.21	6.07	3.59	−5.12	−2.84	2.27	2.01
**2b**	0.66	−1.62	5.76	6.37	6.25	3.48	−5.24	−2.78	2.46	2.28
**2c**	0.14	−1.61	5.24	5.84	5.69	3.49	−4.78	−2.59	2.19	1.75
**2d**	0.76	−1.62	5.86	6.22	6.08	3.48	−5.12	−2.83	2.29	2.38

**Table 2:** Theoretical properties and maximum of experimental absorption and emission of investigated compounds: λ_max_—maximum wavelength of absorption; λ^em^_max_ —maximum wavelength of emission; *ex*—experimental; *cal*—calculated; *f*—oscillator strength.

Compound	λ_max_^ex^ (nm)	λ_max_^cal^ (nm)	*λ^em^_max_* (nm)	*f*
**2a**	355	413 (H-L+1)	609	0.88
	483	623 (H-L)		0.73
**2b**	332	393 (H-L+1)	592	0.86
	468	578 (H-L)		0.69
**2c**	370	418 (H-L+1)	667	0.91
	526	652 (H-L)		0.68
**2d**	311	406 (H-L+1)	563	0.9
	452	619 (H-L)		0.69

**Table 3:** Redox properties of polymers. E^ox^_onse*t*_—oxidation peak onset*;* E^red^_onset_—reduction peak onset*;* IP—ionization potential, estimated from the equation IP [eV] = |e|5.1 + E^ox^_onset_ [V]; EA—electron affinity, estimated from the equation EA [eV] = |e|5.1 + E^red^_onset_ [V]; ΔE_g_^el^ —electrochemical bandgap, from the equation ΔE_g_^el^ = IP-EA.

Compound	E^ox^_onset_ (V)	E^red^_onset_ (V)	IP (eV)	EA (eV)	ΔE_g_^el^ (eV)
**p2a**	0.17	−1.47	5.27	3.63	1.64
**p2b**	0.01	−1.63	5.11	3.47	1.64
**p2c**	−0.69	−1.41	4.41	3.69	0.72
**p2d**	0.09	−1.28	5.19	3.82	1.37

## Data Availability

Data can be made available upon written request to the corresponding author and with a proper justification.

## Sample Availability

Samples of the compounds **2a**–**d** are available from the authors.
